# A Practical Treatise on the Diseases of Children

**Published:** 1847-08

**Authors:** 


### PART III.—BIBLIOGRAPHICAL NOTICES.

ARTICLE IX.

*A Practical Treatise on the Diseases of Children.* By D. Francis Condie, M.D., Secretary of the College of Physicians; Member of the American Philosophical Society; Honorary Member of the Philadelphia Medical Society, etc. Second Edition, Revised and Augmented. Philadelphia: Lea & Blanchard. 1847. 8vo, pp. 657. (From the Publishers. For sale by Joseph Keen, Jr., Chicago.)

The first edition of this work was published in 1844. It having been for three years before the public and adopted as a text book in many of our Medical Institutions, that it has passed to a second edition is good evidence that it is considered a work of value and authoritative on the subjects of which it professes to treat. In the present edition the author has revised the whole work, re-written portions and added such new matter as has been developed since the publication of the first edition. It is now a work that may be consulted with the certainty of obtaining the latest views of the diagnosis, pathology, and treatment of the diseases incident to infancy and childhood. J. V. Z. B.

